# Disease proportions attributable to environment

**DOI:** 10.1186/1476-069X-6-38

**Published:** 2007-11-28

**Authors:** Rodolfo Saracci, Paolo Vineis

**Affiliations:** 1 IFC-National Research Council, via Trieste 41, 56126 Pisa, Italy; 2 Imperial College London, St. Mary's Campus, Norfolk Place W2 1PG, London, UK

## Abstract

Population disease proportions attributable to various causal agents are popular as they present a simplified view of the contribution of each agent to the disease load. However they are only summary figures that may be easily misinterpreted or over-interpreted even when the causal link between an exposure and an effect is well established. This commentary discusses several issues surrounding the estimation of attributable proportions, particularly with reference to environmental causes of cancers, and critically examines two recently published papers. These issues encompass potential biases as well as the very definition of environment and of environmental agent. The latter aspect is not just a semantic question but carries implications for the focus of preventive actions, whether centred on the material and social environment or on single individuals.

## Introduction

Disease proportions attributable to various causal agents are figures popular with scientists, decision makers, and lay people. They are taken, often simplistically, as neat yardsticks to gauge the relative importance of agents on which to direct research or public health efforts. Among Richard Doll's citations, the comprehensive review of the proportions of cancer attributable to various causes [1] ranks first with 1465 quotations well ahead of 1072 hits for the 1994 article reporting the forty-year follow-up study on smoking and health in the British doctors cohort, a cornerstone of modern epidemiology [2].

Attributable proportions are a measure of risk delicate to interpret and potentially misleading. Even when a causal link is well-established between an exposure and an effect (both accurately defined), the population attributable proportion is context dependent in a more involved way than other measures of absolute or relative risk. Two recent publications highlight some of the recurring problems specifically concerning the proportion of diseases attributable to environmental exposures. The first publication by Prüss-Üstün and Corvalan [3] from the World Health Organization expands on the environment section of a previous one [4]. It estimates the etiological contribution of eight classes of environmental factors to eighty five diseases worldwide and in the world macro-regions, variably combining published information, surveyed expert opinion and "ad hoc" calculations. Attributable proportions are derived, with 95% "confidence" limits, and the exercise is prolonged into a calculation of disability-adjusted life years. The second paper [5] by Boffetta et al. from the International Agency of Research on Cancer focuses on one disease group, cancers: it notes the worldwide estimate of 19% of all cancers attributable to environment produced by Prüss-Üstün and Corvalan and argues that this may be a gross overestimation.

## Discussion

### Comparing like with like

Are the two papers using the same definition of environment? Boffetta et al. rightly point out that "ambiguities in the terminology and the inconsistencies in the use of vocabulary by cancer researchers contribute to public confusion regarding the role of environmental causes of cancer". After examining biases potentially leading to overestimation they conclude: "A systematic combination of these errors and bias in a single direction (*reference *[3]*is cited*) may well lead to estimates of cancers attributable to pollutants one order of magnitude larger than the range of reasonably accepted estimates (*references *[1,6,7]*are cited*)". Unfortunately this statement adds to the confusion because no discrepancy of one order of magnitude between the estimates is found when examining the cited sources.

There is instead a substantial difference in the definition of environment, as stressed in a recent letter by Prüss-Üstün and Corvalan [8]. Boffetta et al.'s conclusion is about pollutants which they define as "air, water, soil or food pollutants,..", while Prüss-Üstün and Corvalan define environment as eight broad classes of agents [[3], p.27–28]. They do not provide, however, figures of attributable proportions by disease and class of environmental agents. Indirect information can be found in the related WHO publication [[4], pages 186 and 226]. Out of 56,554,000 deaths there are 3,517,000 deaths *from all diseases *worldwide attributed to the subset of factors in the Prüss-Üstün and Corvalan definition that match Boffetta et al.'s definition (i.e. pollutants): outdoor air pollution; indoor air pollution from solid fuel use; lead; water, sanitation and hygiene (as at least half of the deaths related to the latter agents are reasonably ascribable to micro-organisms rather than to pollutants only half of the deaths attributed to water, sanitation and hygiene are included in the 3,517,000 total for the purpose of the present analysis). This adds-up to 6.2% of all deaths attributable to environmental pollutants, while with the enlarged definition of Prüss-Üstün and Corvalan [[3], page 82] 13,295,000/57,029,000 deaths, i.e. 23.3%, are attributed to the environment (namely 23.3/6.2 = 3.75 times more). If this factor (3.75) is used to adjust down the percentage (19%) of all cancers attributed to environment by the enlarged definition of Prüss-Üstün and Corvalan, a percentage is derived of 5.1% of all cancers attributable to pollutants. This figure is less than twofold the estimate by Doll and Peto [[1], table 11] of about 3% for pollution, food additives and industrial products. Adding to environmental pollutants the carcinogens in the occupational environment an estimate of 5.3% is obtained for Prüss-Üstün and Corvalan against an estimate of 7.0% for Doll and Peto.

These are, if anything, surprisingly tiny differences taking into account: (a) that the scaling down factor derived for all diseases in the whole population is a crude surrogate for disease-specific factors, particularly for occupational agents that affect mostly adult males (this accounts for the small difference between 5.1% and 5.3% when occupational carcinogens are added to pollutants) ; (b) the differences in estimation methods and (c) most relevant, the fact that the Doll and Peto estimate refers to the United States in late 1970's while the Prüss-Üstün and Corvalan's refers to the whole world in the early 2000's. Hence there is a limited difference, not at all of an order of magnitude (tenfold), between the estimates. In fact they may be less independent than they appear at first, as the experts surveyed by Prüss-Üstün and Corvalan were probably well aware of the Doll and Peto pivotal reference.

*Whether the estimates can be regarded as faithfully reflecting today's reality is an altogether different point*; discussing it would first require the actual details for all components of the Prüss-Üstün and Corvalan estimate.

### Methodological issues

Although a unidirectional accumulation of multiple overestimation biases has clearly not materialized in the case just examined – and may be in general infrequent – some of the potential overestimation biases discussed by Boffetta et al. deserve comment.

First attributable proportions may be derived by straightforwardly combining relative risks from studies involving high exposures with figures of exposure prevalence that include people at low exposures. This gross mismatching has been analyzed for almost three decades [1] and awareness should prevent it. In situations in which an exposure-response curve is known, an attributable proportion can be computed for whatever level(s) of exposure reasonably accurate information of the exposure prevalence happens to be available. The result will be in general weakly sensitive to the particular level(s) chosen as in the attributable proportion formula higher relative risks at high exposures tend to be compensated by a lower prevalence while lower relative risks at low exposures tend to be balanced by a higher prevalence.

The exposure-response may, however, be in turn biased, as Boffetta et al note. They argue that the slope of a linear or linearized exposure-response curve may be derived from studies in which past exposures, typically in the occupational environment, have been underestimated: they were in fact higher than estimated through present day reconstruction. If this is the case, the slope of the curve will be biased, i.e. steeper than the true slope, resulting in a spuriously high increase in risk per unit of exposure. As a consequence, any attributable proportion, whatever the exposure level at which is calculated, will be also overestimated.

It is not known how general and sizeable this phenomenon may be, while two other sources of biases tend to act in the opposite direction towards underestimating risk. First, measurements in the occupational environment may have been performed in the past to check compliance with hygiene control limits rather than for epidemiological investigations. For this regulatory purpose sites of presumed high exposure are often selected for measurement, biasing the estimation of the exposure distribution levels towards the high values. As these values may be the only ones available, they may be taken as typical for the whole exposed workforce resulting in an underestimation of the slope of the exposure-response line. Second, some exposure non-differential misclassification is invariably present which tends (in expectation) to make the slope of the exposure-response line shallower than the true one, leading to an underestimate of the attributable proportion. This well known attenuation effect may be substantial for measurement methods with sensitivities in the range 0.60–0.70 and specificities in the range 0.90–0.95, as often is the case for methods employed to assess environmental exposures [9].

Finally, Boffetta et al. appositely recall that exposure-response curves and more generally the effects of one agent can be modified by other agents. Two corollaries follow : (a) attributable proportions may be fully valid when referred to a local and well specified context (a town, a firm etc.) while it may be problematic to gauge their validity when referred to large and heterogeneous population aggregates; this applies particularly to estimates extrapolated from one region or country to another or computed worldwide; (b) because a disease may be attributed simultaneously to more than one cause (e.g. myocardial infarction to blood pressure and tobacco smoking) the sum of attributable proportions is not bound to a 100% maximum. Indeed in an ideal state of full knowledge of causes, not all acting independently, the sum of attributable proportions must exceed 100% (this is probably the most easily forgotten property of attributable proportions).

### Which environment?

From a preventive viewpoint, attributable proportions become regarded as "avoidable proportions" of a disease, except for obviously non-modifiable factors like age or gender. This opens the possibility of grouping agents according to the modes of feasible- or presumptively feasible-modification and this in turn reflects implicit policy choices: should, for instance, tobacco smoking be grouped within a category of "personal behaviours" or within a category of "environmental factors"? In a health policy context, the choice may reflect not only scientific considerations, but also, or even more, the priority placed on different preventive perspectives and approaches. In order to avoid the ambiguities potentially arising from an all encompassing term of "environment" ("all that which is external to the individual human host" [10]) Boffetta et al. propose to abandon it in favour of terms as "non-genetic" and "pollutants". From the preventive viewpoint this is a dangerous proposal, all the more as it comes from an authoritative organisation, the International Agency for Research on Cancer. Words are not neutral: whatever the good intentions evacuating the term "environment" from the etiological lexicon would concentrate all attention on the exposed individual and on his/her almost exclusive responsibility in hazard prevention, an undesirable shift just at a time when individual susceptibility already over-occupies the forefront of etiological research. The terms "environment" and "environmental" should remain, with detailed descriptors accurately defining the specific components of the environment.

## Conclusion

A general consideration underpins the comments just presented: population attributable proportions of a disease are summary indexes attractive for their simplicity but subject to severe limitations.

For *descriptive purposes *it should be born in mind that they are relative figures. A small attributable proportion in a population with a high incidence of a disease may in fact reflect a higher incidence rate – the basic expression of causal factors – than a high proportion in another population with a low incidence.

For *etiological research purposes *the population attributable proportion is of no use as such. It needs to be decomposed into its two elements, exposure prevalence and attributable fraction among the exposed: only the latter tells how much an exposure under consideration accounts for the disease (assuming causality is established), hence how worth it may be to look for other causes as well.

Finally for *public health purposes *the population attributable proportion says nothing of the actual preventability of a cause in its technical, social, economic, psychological and ethical dimensions. When these factors are not explicitly and analytically taken into account, the attributable proportions may easily be misused as ranking tools to establish a "preventable cause's league" instead of being employed, as they should, simply as pointers to a cause impact. Impact is only one element in deciding where to concentrate public health actions and/or research on preventive tools. Confusion is added if exposures belonging to the environment, i.e. external to the individual, are outlined only in generic and broad terms rather than be accurately specified and their causal role corroborated before they are grouped into larger classes (causality criteria for macro-environmental exposures like "global climate change" pose however special problems beyond the scope of the present discussion).

It is worth stressing that from a population-based public health perspective, causes recognized as environmental call primarily for interventions on the material and social environment ahead of measures addressed to single individuals.

## Competing interests

The author(s) declare that they have no competing interests.

## Authors' contributions

Both authors contributed to the review of the papers that prompted the commentary, to the identification of key discussion items and to the writing and revision of the text.
